# Light‐Addressable Nanoclusters of Ultrasmall Iron Oxide Nanoparticles for Enhanced and Dynamic Magnetic Resonance Imaging of Arthritis

**DOI:** 10.1002/advs.201901800

**Published:** 2019-08-08

**Authors:** Xin Li, Shiyi Lu, Zuogang Xiong, Yong Hu, Dan Ma, Wenqi Lou, Chen Peng, Mingwu Shen, Xiangyang Shi

**Affiliations:** ^1^ State Key Laboratory for Modification of Chemical Fibers and Polymer Materials International Joint Laboratory for Advanced Fiber and Low‐Dimension Materials College of Chemistry Chemical Engineering and Biotechnology Donghua University Shanghai 201620 P. R. China; ^2^ Cancer Center, China‐France Joint Laboratory for Healthcare Theranostics Shanghai Tenth People's Hospital Tongji University School of Medicine Shanghai 200072 P. R. China; ^3^ Ninghai First Hospital Ningbo 315600 P. R. China

**Keywords:** folic acid‐mediated targeting, inflammatory arthritis, light‐addressable nanoclusters, *T*_1_/*T*_2_‐weighted MR imaging, ultrasmall Fe_3_O_4_ NPs

## Abstract

Design of novel nanoplatforms with single imaging elements for dynamic and enhanced *T*_1_/*T*_2_‐weighted magnetic resonance (MR) imaging of diseases still remains significantly challenging. Here, a facile strategy to synthesize light‐addressable ultrasmall Fe_3_O_4_ nanoparticles (NPs) that can form nanoclusters (NCs) under laser irradiation for enhanced and dynamic *T*_1_/*T*_2_‐weighted MR imaging of inflammatory arthritis is reported. Citric acid‐stabilized ultrasmall Fe_3_O_4_ NPs synthesized via a solvothermal approach are linked with both the arthritis targeting ligand folic acid (FA) and light‐addressable unit diazirine (DA) via polyethylene glycol (PEG) spacer. The formed ultrasmall Fe_3_O_4_‐PEG‐(DA)‐FA NPs are cytocompatible, display FA‐mediated targeting specificity to arthritis‐associated macrophage cells, and can form NCs upon laser irradiation to have tunable *r*_1_ and *r*_2_ relaxivities by varying the laser irradiation duration. With these properties owned, the designed Fe_3_O_4_‐PEG‐(DA)‐FA NPs can be used for *T*_1_‐weighted MR imaging of arthritis without lasers and enhanced dual‐mode *T*_1_/*T*_2_‐weighted MR imaging of arthritis under laser irradiation due to the formation of NCs that have extended accumulation within the arthritis region and limited intravasation back to the blood circulation. The designed light‐addressable Fe_3_O_4_‐PEG‐(DA)‐FA NPs may be used as a promising platform for dynamic and precision *T*_1_/*T*_2_‐weighted MR imaging of other diseases.

Inflammatory arthritis is one of the most common diseases affecting human health because of its high incidence and disability rate of up to 50% in the patients with an age of over 50.^1^ Although many techniques have been developed for the diagnosis of arthritis such as biomarkers, computed tomography, and ultrasonic imaging, their efficacy for patients at an advanced stage is not satisfactory.^2^ As such, development of multifunctional nanoplatforms for early diagnosis of arthritis still remains an urgent challenge. Until now, noninvasive magnetic resonance (MR) imaging with high spatial resolution has been considered to be a powerful technique for early detection of arthritis.^3^

In general, to improve the sensitivity and accuracy of diagnosis, multifunctional contrast agents with dual‐mode or multimode imaging elements integrated have been designed.^4^ In particular, for dual‐mode *T*_1_/*T*_2_‐weighted MR imaging, the reported contrast agents are usually composed of two types of imaging agents, such as gadolinium‐ or manganese‐based NPs (*T*_1_ positive agents) combined with superparamagnetic iron oxide (Fe_3_O_4_) NPs (*T*_2_ negative agents) within or onto a single nanosystem.^5^ However, the strategy used for simple combination of two types of functional elements often leads to unsatisfactory results,^6^ due to the existing issues of colloidal instability, the composition‐dependent *r*_1_/*r*_2_ ratio, and the difficulty for further surface functionalization. Furthermore, it is difficult to achieve the tunable conversion or coexistence of *T*_1_‐weighted and *T*_2_‐weighted MR imaging capability based on one kind of NPs for the clinical applications.

Ultrasmall Fe_3_O_4_ NPs with a size smaller than 5 nm have been reported to have *T*_1_ enhancing effect and *T*_2_ decreasing effect, and can be used as an excellent contrast agent for *T*_1_‐weighted MR imaging.^7^ Through assembly of ultrasmall Fe_3_O_4_ NPs onto generation 5 (G5) poly(amidoaimine) dendrimers, the generated nanoclusters can be readily developed as a contrast agent for *T*_2_‐weighted MR imaging of tumors.[qv: 7a] Recent reports have also shown that ultrasmall Fe_3_O_4_ NPs can be assembled to form larger clusters in a suitable environment (e.g., specific pH or enzyme) and realize the conversion from *T*_1_‐weighted to *T*_2_‐weighted MR imaging.^8^ However, these approaches using pH‐ or enzyme‐responsive assembly may generate unwanted particle aggregation due to the sophisticated biological environment that is susceptible to nucleases in blood and may further induce immune response after particle exposure.^9^ Additionally, the pH‐ or enzyme‐based responsive assembly of particles mostly occurs in a few types of tumor microenvironment,[qv: 8b,10] and may not be suitable for the design of responsive assembly of ultrasmall Fe_3_O_4_ NPs for precision imaging of other diseases, in particular inflammatory arthritis. Moreover, a part of NPs with a small size are capable of escaping the rapid renal clearance, easily extravasate from vessels around lesion, and subsequently penetrate into the lesion region. However, these NPs also readily intravasate back into circulation, leading to decreased accumulation in the lesion.^11^ Therefore, it is vital to realize the enhanced retention of particles and obtain precise dynamic dual‐mode *T*_1_/*T*_2_‐weighted MR imaging of arthritis in vivo based on the ultrasmall Fe_3_O_4_ NPs. In particular, light‐triggered assembly and hierarchical targeting^12^ may be used as an efficient strategy to bypass some hurdles because of most of the diseases (e.g., inflammation, tumors, etc.) can be spatiotemporally addressed by light.^13^

In our previous work, we synthesized G5 dendrimer‐stabilized gold (Au) nanoflowers embedded with ultrasmall Fe_3_O_4_ NPs.^14^ The designed hybrid nanoplatform possessed a higher *r*_1_ relaxivity of 3.22 mm ^−1^ s^−1^ by embedding ultrasmall Fe_3_O_4_ NPs within Au nanoflowers with a well distribution and limited aggregation. In another work, Gao and co‐workers designed light‐triggered Au NP‐based nanoclusters that enable photothermal therapy and photoacoustic imaging of tumors in vivo,[qv: 13a] where the Au NPs are able to be clustered via a polyethylene glycol (PEG)‐linked diazirine (DA) terminal group under laser exposure. These prior works stimulate us to hypothesize that by reasonable adjustment of the aggregation degree of the ultrasmall Fe_3_O_4_ NPs through spatiotemporal manipulation, the NPs may be used as an excellent nanoprobe for dynamic dual‐mode *T*_1_/*T*_2_‐weighted MR imaging of arthritis.

In this present work, we designed light‐addressable assemblies of ultrasmall Fe_3_O_4_ NPs for enhanced retention and dynamic *T*_1_/*T*_2_‐weighted MR imaging of inflammatory arthritis in vivo. First, ultrasmall citric acid‐stabilized Fe_3_O_4_ NPs were synthesized via a solvothermal method, and modified with arthritis‐targeting ligand folic acid (FA)^15^ and molecular switch DA via a PEG spacer through 1‐ethyl‐3‐(3‐dimethylaminopropyl) carbodiimide hydrochloride (EDC) coupling reaction. The linked molecular switch DA on the surface of Fe_3_O_4_‐PEG‐(DA)‐FA NPs was transformed to carbene under 405 nm laser excitation, which was further reacted with adjacent ligands onto the Fe_3_O_4_ NPs to form covalent bonds of C—C, C—H, O—H, and X—H (X represents heteroatom), leading to the formation of nanoclusters (NCs) that were used for dynamic light‐addressable *T*_1_/*T*_2_‐weighted MR imaging of arthritis (**Figure** **1**). The main idea to be tested was as follows: After intravenous delivery, the Fe_3_O_4_‐PEG‐(DA)‐FA NPs can easily extravasate through the vasculature around arthritis and subsequently penetrate inside the inflammation region, allowing for *T*_1_‐weighted MR imaging of the arthritis. After 405 nm laser irradiation to induce the formation of NCs in the inflammation region, the formed NCs are not able to intravasate back into circulation and remain in the inflammation region of arthritis, thus allowing for enhanced *T*_1_/*T*_2_‐weighted dual‐mode MR imaging. We systematically characterized the materials, tested their cytocompatibility, FA‐mediated targeting specificity, and dynamic light‐addressable MR imaging performance of arthritis in vivo. To our knowledge, this is the very first example to design an FA‐targeted, light‐addressable nanoplatform that can be used for dynamic dual‐mode *T*_1_/*T*_2_‐weighted precision MR imaging of arthritis.

**Figure 1:**
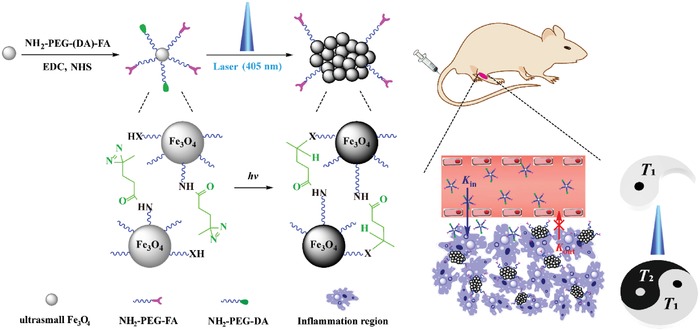
Schematic illustration of the synthesis of Fe_3_O_4_‐PEG‐(DA)‐FA NPs for enhanced retention and tunable *T*_1_/*T*_2_‐weighted MR imaging of inflammatory arthritis.

To prepare light‐addressable NCs composed of ultrasmall Fe_3_O_4_ NPs, we first synthesized citric acid‐stabilized ultrasmall Fe_3_O_4_ NPs with an average diameter of 2.8 nm via a solvothermal method. The size and uniform morphology of the particles were characterized and confirmed by transmission electron microscopy (TEM, Figure S1, Supporting Information). We then synthesized NHS‐DA, which was characterized by ^13^C NMR and ^1^H NMR, respectively (Figure S2, Supporting Information). Next, NH_2_‐PEG‐NHBoc was simultaneously modified with NHS‐DA and NHS‐FA, and deprotected by hydrochloric acid. The formed mixture of NH_2_‐PEG‐(DA)‐FA was characterized by ^1^H NMR (Figure S3, Supporting Information). Through NMR integration, the numbers of DA and FA conjugated to each PEG were estimated to be 0.68 and 0.26, respectively.

After that, NH_2_‐PEG‐(DA)‐FA was immobilized onto the surface of citric acid‐stabilized ultrasmall Fe_3_O_4_ NPs via EDC chemistry, leading to the formation of Fe_3_O_4_‐PEG‐(DA)‐FA NPs. The amount of NH_2_‐PEG‐(DA)‐FA modified onto the surface of the Fe_3_O_4_ NPs was quantified by thermogravimetric analysis (TGA, Figure S4, Supporting Information). Based on the weight loss data, the percentage of PEG‐(DA)‐FA modified onto the particle surface can be calculated to be 14.3%. Inductively coupled plasma‐optical emission spectroscopy (ICP‐OES) measurements were used to quantify the Fe element within the Fe_3_O_4_‐PEG‐(DA)‐FA NPs and the Fe content was quantified to be 220 µg mg^−1^.

The light‐responsive aggregation of the Fe_3_O_4_‐PEG‐(DA)‐FA NPs was investigated via TEM imaging (**Figure** **2**). Before laser irradiation, the Fe_3_O_4_‐PEG‐(DA)‐FA NPs display a certain degree of aggregation, which is likely due to the sample drying process of the PEGylated ultrasmall Fe_3_O_4_ NPs. After irradiated by a 405 nm laser (1.0 W cm^−2^) for different time periods, the aggregation of the NPs is apparent (Figure 2a). The aggregation degree of the NPs is dependent on the laser irradiation duration. The formed Fe_3_O_4_‐PEG‐(DA)‐FA NCs comprise of many single Fe_3_O_4_‐PEG‐(DA)‐FA NPs with anisotropic crystalline orientations and the crystal structure and composition of the ultrasmall Fe_3_O_4_ NPs have not changed upon laser irradiation for different time periods (inset of Figure 2a,b). The laser irradiation time‐dependent aggregation of the Fe_3_O_4_‐PEG‐(DA)‐FA NPs was further confirmed by dynamic light scattering measurements (Figure 2c), where the hydrodynamic size of the Fe_3_O_4_‐PEG‐(DA)‐FA NPs increases as a function of laser irradiation time. The Fe_3_O_4_‐PEG‐(DA)‐FA NPs exhibit an initial hydrodynamic size of around 45.7 nm, and after laser irradiation for 12 min eventually reach a size up to 798.4 nm. Further, the clear aggregation of Fe_3_O_4_‐PEG‐(DA)‐FA NCs can be observed in aqueous solution after laser irradiation for 12 min (Figure S5, Supporting Information), and the aggregates can be redispersed to form a stable solution after shaking. This indicates that the light‐addressable DA terminal groups enable the self‐crosslinking of the particles under laser irradiation, likely via the formation of covalent bonds of C—C, C—H, O—H, and X—H (X represents heteroatom) between the formed DA carbine and adjacent ligands onto the Fe_3_O_4_ NPs, in agreement with the literature.[qv: 13a] Moreover, the agglomeration degree of Fe_3_O_4_‐PEG‐(DA)‐FA NCs could be precisely controlled through variation of the laser irradiation time period.

**Figure 2:**
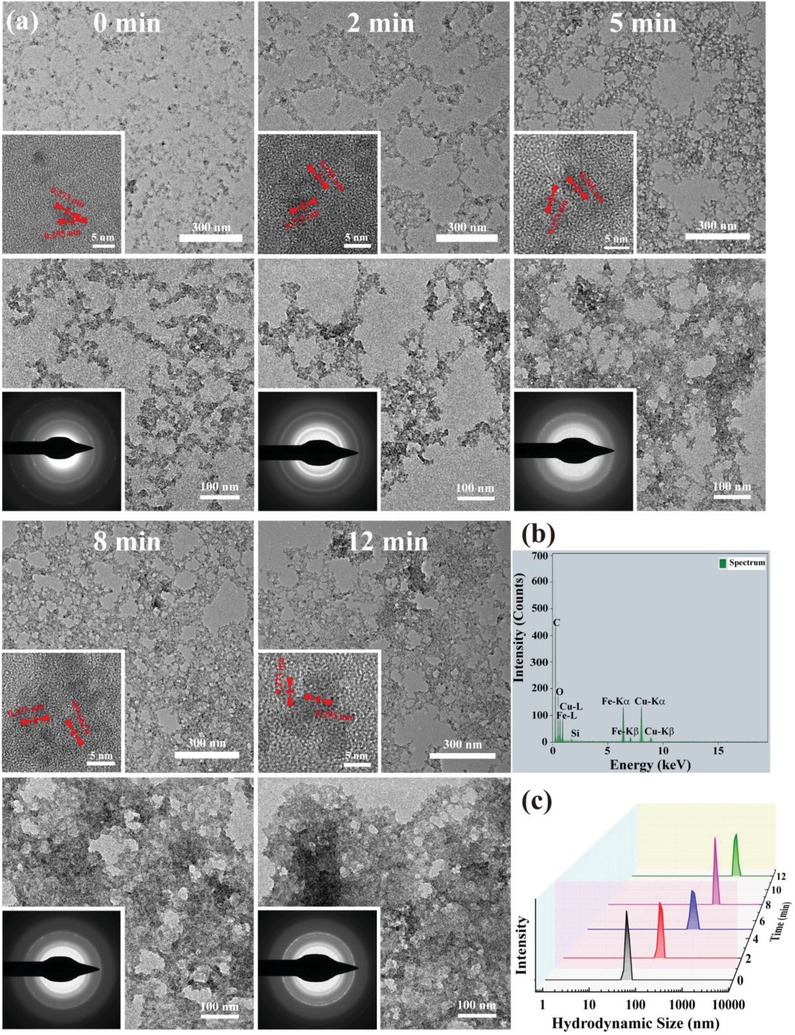
a) TEM images, b) energy‐dispersive X‐ray spectroscopy, and c) hydrodynamic size of Fe_3_O_4_‐PEG‐(DA)‐FA NPs under 405 nm laser irradiation (1.0 W cm^−2^) for different periods of time.

The MR relaxometry of the Fe_3_O_4_‐PEG‐(DA)‐FA NPs and NCs formed after laser irradiation was next explored. The *T*_1_ and *T*_2_ relaxation rate of the particles as a function of laser irradiation time period was determined (**Figure** **3**a,b). The *r*_1_ and *r*_2_ relaxivities of Fe_3_O_4_‐PEG‐(DA)‐FA NCs are size‐dependent and can be precisely tuned by different laser irradiation durations. The initial Fe_3_O_4_‐PEG‐(DA)‐FA NPs possess a high *r*_1_ relaxivity (3.83 mm ^−1^ s^−1^) and low *r*_2_ relaxivity (9.04 mm ^−1^ s^−1^). After laser irradiation for 12 min, their *r*_1_ and *r*_2_ relaxivities change to 1.61 and 31.6 mm ^−1^ s^−1^, respectively. It is worth mentioning that the developed Fe_3_O_4_‐PEG‐(DA)‐FA NPs possess a higher *r*_1_ relaxivity than that of ultrasmall Fe_3_O_4_ NPs (about 1.2–1.4 mm ^−1^ s^−1^) in previous reports.^7^ This may be due to the fact that the PEGylation modification along with the DA and FA functional groups may enable them to form a certain degree of aggregation with extended interparticle distance, thereby enhancing their *r*_1_ relaxivity, in consistence with our previous work.^14^

**Figure 3:**
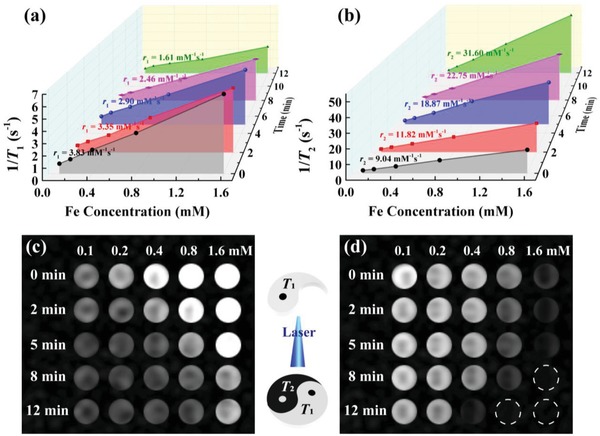
a) *T*_1_ relaxation rates, b) *T*_2_ relaxation rates, and c) *T*_1_‐weighted MR imaging, d) *T*_2_‐weighted MR imaging as a function of Fe concentration for the Fe_3_O_4_‐PEG‐(DA)‐FA NPs and Fe_3_O_4_‐PEG‐(DA)‐FA NCs under 405 nm laser irradiation (1.0 W cm^−2^) for different periods of time.

Furthermore, the change of the *r*_1_ and *r*_2_ relaxivities can be reflected by the switch between *T*_1_ effect (hyperintensity contrast) and *T*_2_ effect (hypointensity contrast) of the Fe_3_O_4_‐PEG‐(DA)‐FA NCs (Figure 3c,d and Figure S6, Supporting Information). The Fe_3_O_4_‐PEG‐(DA)‐FA NPs only possess hyperintensity of *T*_1_ effect due to their high *r*_1_ relaxivity and low *r*_2_ relaxivity. Interestingly, the intensity of *T*_1_ effect of Fe_3_O_4_‐PEG‐(DA)‐FA NCs gradually decreases with the time of laser irradiation (0–12 min), while the intensity of *T*_2_ effect of Fe_3_O_4_‐PEG‐(DA)‐FA NCs gradually increases with the time of laser irradiation at the same Fe concentrations. The Fe_3_O_4_‐PEG‐(DA)‐FA NPs and Fe_3_O_4_‐PEG‐(DA)‐FA NCs after laser irradiation for 2 min exhibit the excellent *T*_1_ effect, while the Fe_3_O_4_‐PEG‐(DA)‐FA NCs after laser irradiation for 8 and 12 min turn to have the stronger *T*_2_ effect. This means that the Fe_3_O_4_‐PEG‐(DA)‐FA NPs after laser irradiation exhibit a clear switch from *T*_1_ to *T*_2_ effect and the switch process is precisely tuned through variation of laser irradiation time. The reasons for the gradual decrease of *r*_1_ relaxivity and increase of *r*_2_ relaxivity of the Fe_3_O_4_‐PEG‐(DA)‐FA NPs after laser irradiation may stem from the reduced interparticle distance after the crosslinking reaction and simultaneous increase of the mass magnetization values of the Fe_3_O_4_ NPs.^16^ In a recent work, Dullens and co‐workers synthesized superparamagnetic nickel colloidal NCs displaying the property of tunable size and mass magnetization by a spontaneous self‐organization procedure, and their results also supported our conclusion.^17^ In addition, the merits of Fe_3_O_4_‐PEG‐(DA)‐FA NCs are remarkable since the agglomeration degree of NCs can be precisely tuned through the external laser irradiation, hence the retention of the particles in the disease site may be controlled by changing the size of the aggregated NPs.

In order to further understand the *T*_1_–*T*_2_ switching property of the Fe_3_O_4_‐PEG‐(DA)‐FA NPs, we calculated the *r*_2_/*r*_1_ ratios of the NPs after laser irradiation (Figure S7, Supporting Information) to explain the competition of *T*_1_ and *T*_2_ effect according to theory[qv: 4c] and the literature,^18^ since the *r*_2_/*r*_1_ ratio is an important parameter widely used for evaluating the *T*_1_ or *T*_2_ effect of Fe_3_O_4_ NPs.^19^ It is quite obvious that the laser irradiation time has strongly impacted the *r_1_*, *r*_2_, and *r*_2_/*r*_1_ ratio. In general, the small Fe_3_O_4_ NPs with a high *r*_1_ relaxivity (>3.0 mm ^−1^ s^−1^) and low *r*_2_/*r*_1_ ratio (<5) show strong *T*_1_ effect and do not show obvious *T*_2_ effect.^20^ In addition, the large Fe_3_O_4_ NPs with a high *r*_2_ relaxivity and *r*_2_/*r*_1_ ratio (more than 25) result in a dominant *T*_2_ effect overwhelming the *T*_1_ effect.[qv: 4c,8b] Interestingly, the relatively high *r*_1_ (1.61 mm ^−1^ s^−1^) and *r*_2_ (31.60 mm ^−1^ s^−1^) relaxivities of the Fe_3_O_4_‐PEG‐(DA)‐FA NCs formed after laser irradiation for 12 min are suitable to be used for potential dual‐mode *T*_1_/*T*_2_‐weighted MR imaging applications. The Fe_3_O_4_‐PEG‐(DA)‐FA NPs having a low *r*_2_ relaxivity (9.04 mm ^−1^ s^−1^) and *r*_2_/*r*_1_ ratio (2.36) can realize a sharp switch from *T*_1_ to *T*_2_ effect after formation of NCs with a larger size under laser irradiation. Meanwhile, the agglomeration degree and *r*_2_/*r*_1_ ratio of Fe_3_O_4_‐PEG‐(DA)‐FA NCs can be precisely controlled by varying the laser irradiation time period, providing a powerful platform for accurate dynamic dual‐mode *T*_1_/*T*_2_‐weighted MR imaging applications.

Before in vivo applications, we first tested the cytotoxicity of the Fe_3_O_4_‐PEG‐(DA)‐FA NPs and evaluated the FA‐mediated targeting specificity of the particles to arthritis‐associated macrophage cells. CCK8 cell proliferation assay data (Figure S8, Supporting Information) reveal that the viability of Raw264.7 cells treated with the Fe_3_O_4_‐PEG‐(DA)‐FA NPs only has slight changes and can maintain above 86.1% in the studied concentration range ([Fe] = 0.2–3.0 × 10^−3^ m) when compared to control cells treated with phosphate buffered saline (PBS). This implies that the Fe_3_O_4_‐PEG‐(DA)‐FA NPs display excellent cytocompatibility. In order to validate the FA‐mediated specific targeting of Fe_3_O_4_‐PEG‐(DA)‐FA NPs to arthritis‐associated macrophage cells, we quantitatively analyzed the Fe uptake by Raw264.7 via ICP‐OES, and the free FA‐blocked cells were also tested under the same conditions (Figure S9, Supporting Information). It can be seen that with the increase of Fe concentration, the Fe uptake by either Raw264.7 cells or free FA‐blocked Raw264.7 cells gradually increases. The Fe uptake by the Raw264.7 cells is about 1.66–1.81 times higher than that by the free FA‐blocked Raw264.7 cells at the Fe concentration range of 0.2–3.0 × 10^−3^ m (*p* < 0.01), suggesting that the attached FA ligand onto the particle surface can actually mediate the particle targeting to macrophage cells expressing FA receptors. The targeting specificity and light‐addressable assembly of the Fe_3_O_4_‐PEG‐(DA)‐FA NPs was further validated by TEM imaging in vitro (Figure S10, Supporting Information). It is apparent that the Fe_3_O_4_‐PEG‐(DA)‐FA NPs are more significantly taken up by Raw264.7 cells than by free FA‐blocked Raw264.7 cells after incubation for 12 h. Meanwhile, the Fe_3_O_4_‐PEG‐(DA)‐FA NCs are formed in the cytoplasm of Raw264.7 cells after exposure to 405 nm laser (1.0 W cm^−2^) for 3 min, while the Fe_3_O_4_‐PEG‐(DA)‐FA NPs without laser irradiation do not seem to have the NCs composed of the aggregated Fe_3_O_4_‐PEG‐(DA)‐FA NPs. These results demonstrate that the modification of FA renders the Fe_3_O_4_‐PEG‐(DA)‐FA NPs with targeting specificity to FA receptor‐expressing macrophage cells and the light‐triggered assembly of Fe_3_O_4_‐PEG‐(DA)‐FA NCs can be realized in vitro.

We next explored the potential to use the Fe_3_O_4_‐PEG‐(DA)‐FA NPs for targeted *T*_1_‐weighted and dual‐mode *T*_1_/*T*_2_‐weighted MR imaging of inflammatory arthritis in vivo, respectively. Animal experiments were carried out following the protocols approved by the institutional committee for animal care and the policy of the National Ministry of Health. For the targeted *T*_1_‐weighted MR imaging, the *T*_1_‐weighted MR images (**Figure** **4**a,b) and corresponding *T*_1_ MR signal‐to‐noise ratio (SNR) (Figure 4c,d) were collected from arthritis and free FA‐blocked arthritis model at different time points postinjection of the Fe_3_O_4_‐PEG‐(DA)‐FA NPs, respectively. It can be seen that for the free FA‐blocked arthritis model, the MR signal intensity of the arthritis region is the highest at 30 min postinjection and decreases with the time postinjection. Excitingly, for the regular arthritis model, the strong MR signal intensity can be lasted from 30 to 60 min postinjection and the MR signal can be still detectable even at 120 min postinjection (Figure 4a and Figure S11, Supporting Information). Quantitative MR SNR data show that the MR SNR of the regular arthritis model is about 1.79–2.66 times higher than that of the free FA‐blocked arthritis model at 15–60 min postinjection (Figure 4c,d). These results indicated that with the FA‐mediated targeting, the designed Fe_3_O_4_‐PEG‐(DA)‐FA NPs enable enhanced accumulation and prolonged residence in the arthritis region, thereby allowing for enhanced *T*_1_‐weighted MR imaging of arthritis.

**Figure 4:**
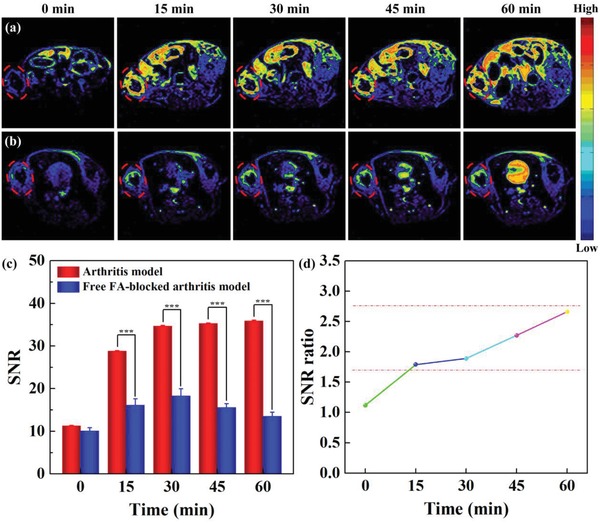
a,b) In vivo *T*_1_‐weighted MR imaging of regular arthritis and free FA‐blocked arthritis model. Dashed line of ellipse represents the arthritis region. c) Corresponding MR SNR of the arthritis region before and at different time points post intravenous injection of the Fe_3_O_4_‐PEG‐(DA)‐FA NPs. d) The MR SNR ratios between arthritis and free FA‐blocked arthritis model before and at different time points post intravenous injection of the Fe_3_O_4_‐PEG‐(DA)‐FA NPs.

In order to avoid the individual differences of mice that influence the targeted *T*_1_/*T*_2_‐weighted MR imaging, the arthritis models in both left and right hind legs for each mouse were established. Then, the *T*_1_/*T*_2_‐weighted MR images (**Figure** **5**a,b) and corresponding MR SNR (Figure 5c,d) of the arthritis model were collected and calculated. The laser irradiation of arthritis region in the left hind leg (red arrow) was carried out, while the arthritis region of right hind leg (white arrow) received no laser irradiation. Clearly, for the *T*_1_‐weighted MR imaging, the arthritis region of both hind legs shows a significant MR contrast enhancement at 30 min postinjection of the Fe_3_O_4_‐PEG‐(DA)‐FA NPs before laser irradiation, which is much higher than that before injection (*p* < 0.001, Figure 5a,c). As expected, the signal intensity and MR SNR of arthritis region in left hind leg after laser irradiation slightly decrease, while the *T*_1_ MR signal intensity of arthritis region in right hind leg without laser irradiation does not have any prominent changes (*p* > 0.05, ns). The MR signal intensity and SNR of left hind leg after laser irradiation are always higher than that of the control (*p* < 0.01). These results demonstrate that the formed Fe_3_O_4_‐PEG‐(DA)‐FA NCs after laser irradiation retain their *T*_1_ contrast capability for *T*_1_‐weighted MR imaging of arthritis.

**Figure 5:**
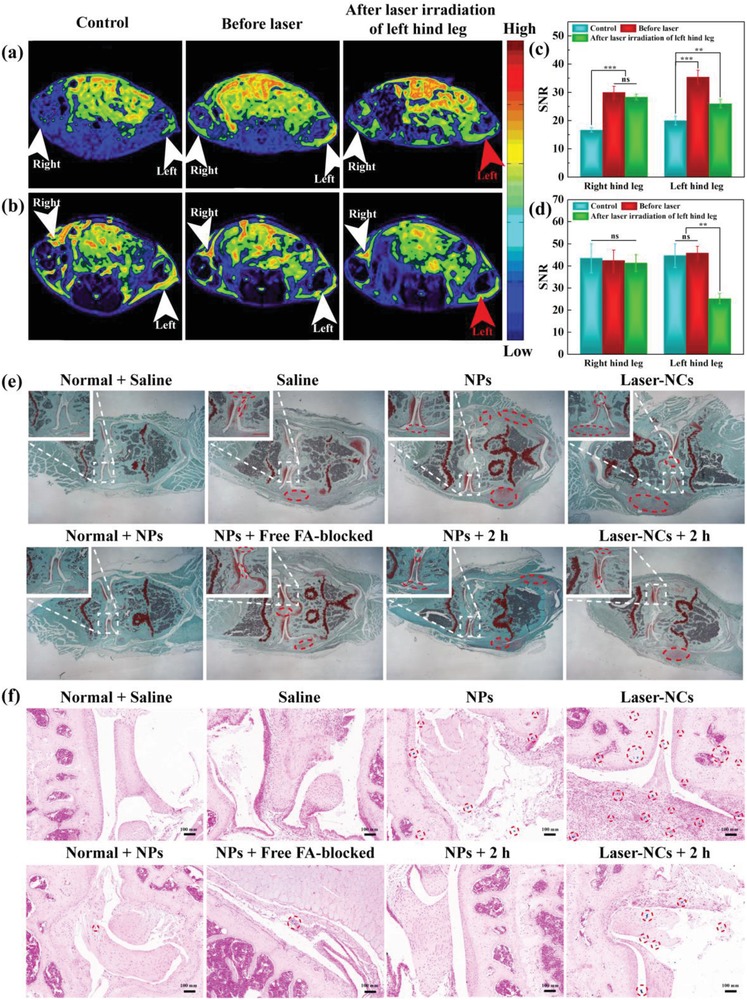
In vivo a) *T*_1_‐weighted, b) *T*_2_‐weighted MR imaging, and c,d) the corresponding MR SNR of arthritis model before (control) and after intravenous injection of Fe_3_O_4_‐PEG‐(DA)‐FA NPs (before and after laser irradiation). The white and red arrows indicate the arthritis region without and with laser irradiation, respectively. e,f) Safranin O and Prussian blue‐stained tissue section of inflammatory arthritis after different treatments. Inset in each panel of (e) shows the magnified region of arthritis. In (f), the red circle indicates the blue staining area.

For *T*_2_‐weighted MR imaging, the arthritis region of both hind legs display similar MR signal intensity for control mouse before injection and at 30 min postinjection of the Fe_3_O_4_‐PEG‐(DA)‐FA NPs before laser irradiation (*p* > 0.05, ns, Figure 5b,d), implying that the Fe_3_O_4_‐PEG‐(DA)‐FA NPs have no *T*_2_‐weighted MR imaging effect. In contrast, the MR signal intensity and SNR value of arthritis region in left hind leg after laser irradiation significantly decrease (*p* < 0.01), which is opposed to the arthritis region in right hind leg without laser irradiation (*p* > 0.05, ns). It is apparent that the aggregated Fe_3_O_4_‐PEG‐(DA)‐FA NCs after laser irradiation can exert their *T*_2_ contrast effect in the arthritis. Overall, the aggregated Fe_3_O_4_‐PEG‐(DA)‐FA NCs after laser irradiation for 12 min can be used as a precision diagnosis nanoprobe for targeted dual‐mode *T*_1_/*T*_2_‐weighted MR imaging of arthritis.

We have distinctly validated that the modification of FA renders the Fe_3_O_4_‐PEG‐(DA)‐FA NPs with specific accumulation in vitro and prolonged residence time of arthritis region in vivo through MR imaging. Further, the enhanced retention and efficient accumulation of aggregated Fe_3_O_4_‐PEG‐(DA)‐FA NCs after laser irradiation in the arthritis region in vivo were investigated by Prussian blue staining (Figure 5f). According to the results of Safranin O and hematoxylin and eosin (H&E) staining (Figure 5e and Figure S12, Supporting Information), the knee joint region of the mice with arthritis model shows some large area of inflammation (dashed red circles or ellipses) when compared to normal mice. For Prussian blue staining, the mice with arthritis model and normal mice in saline groups do not display any blue staining. The normal mice in NPs group and the mice with NPs injected to free FA‐blocked arthritis model group display slight blue staining. In contrast, the arthritis model mice injected with NPs (NPs group) and the further laser irradiation to generate NCs (Laser‐NCs group) exhibit much larger blue areas (red circles) than the above groups, and the blue staining areas of the inflammation region in NCs groups are much larger than those of NPs groups. This indicates that the NPs with ultrasmall size and FA‐mediated targeting specificity could effectively penetrate into the inflammation region, and upon laser irradiation, the NCs are able to better retain in the inflammatory region. After feeding the mice for additional 2 h, the blue color of the stained inflammation region disappears for the NPs + 2 h group because of the fast metabolism of NPs in the mice, whereas the Laser‐NCs + 2 h group still maintains a relatively large blue staining area. These results are in consistence with the results of *T*_1_‐weighted and *T*_1_/*T*_2_‐weighted MR imaging data, suggesting that laser irradiation greatly enhances the retention of NCs with a large size in the arthritis regions. Our study can readily prove our design concept: the small Fe_3_O_4_‐PEG‐(DA)‐FA NPs can not only extravasate from the blood vessels to interstitial space of lesion region but also readily intravasate back into circulation system; upon laser irradiation to generate larger NCs, the NCs sufficiently retained in the lesion region hardly intravasate back to the blood vessels,[qv: 8b,13a] thereby enabled enhanced dual‐mode *T*_1_/*T*_2_‐weighted MR imaging. Overall, the efficient penetration and accumulation in the arthritis are attributed to the ultrasmall size and FA‐mediated specific targeting of the Fe_3_O_4_‐PEG‐(DA)‐FA NPs, and the aggregated Fe_3_O_4_‐PEG‐(DA)‐FA NCs after laser irradiation display enhanced retention in arthritis due to the increased size, in agreement with our hypothesis (*K*_in_ ≫ *K*_out_) described in Figure 1.

In summary, we present a facile method to generate light‐addressable Fe_3_O_4_‐PEG‐(DA)‐FA NPs that can be used as a novel nanoplatform for dynamic enhanced dual‐mode *T*_1_/*T*_2_‐weighted MR imaging of arthritis. We show that through EDC coupling chemistry, ultrasmall citric acid‐stabilized Fe_3_O_4_ NPs can be modified with targeting ligand FA and light‐addressable unit DA via PEG spacer. The generated Fe_3_O_4_‐PEG‐(DA)‐FA NPs display good cytocompatibility and targeting specificity to arthritis‐associated macrophage cells, and can be used for *T*_1_‐weighted MR imaging of arthritis due to their high *r*_1_ relaxivity (3.83 mm ^−1^ s^−1^). Upon laser irradiation, NCs of the Fe_3_O_4_‐PEG‐(DA)‐FA NPs can be formed to have tunable aggregation degree and hence tunable *r*_1_ (from 3.83 to 1.61 mm ^−1^ s^−1^) and *r*_2_ (9.04 to 31.6 mm ^−1^ s^−1^) relaxivities via variation of laser irradiation duration. The optimized NCs of Fe_3_O_4_‐PEG‐(DA)‐FA NPs having strong *T*_2_ effect and noncompromised *T*_1_ effect are able to be used for enhanced dual‐mode *T*_1_/*T*_2_‐weighted MR imaging of inflammatory arthritis presumably due to the fact that the easily extravasated individual Fe_3_O_4_‐PEG‐(DA)‐FA NPs are able to be located in the arthritis region, and form NCs upon laser irradiation that are difficult to intravasate back to the blood circulation. The designed light‐addressable Fe_3_O_4_‐PEG‐(DA)‐FA NPs may be used as a promising nanoplatform for dynamic and enhanced *T*_1_/*T*_2_‐weighted dual‐mode MR imaging of other diseases (e.g., cancer) with high precision.

## Conflict of Interest

The authors declare no conflict of interest.

## Supporting information

Supplementary
