# Supplementary material for: Light‐Addressable Nanoclusters of Ultrasmall Iron Oxide Nanoparticles for Enhanced and Dynamic Magnetic Resonance Imaging of Arthritis
Source: Adv Sci (Weinh). 2019 Aug 8;6(19):1901800. doi: 10.1002/advs.201901800 (PMC6774037; doi:10.1002/advs.201901800)
Supplement: Supplementary file 1 — Supplementary [file ADVS-6-1901800-s001.pdf]

Copyright WILEY-VCH Verlag GmbH & Co. KGaA, 69469 Weinheim, Germany, 2019.

ADVANCED SCIENCE
Open Access

Supporting Information

for *Adv. Sci.,* DOI: 10.1002/advs.201901800

Light-Addressable Nanoclusters of Ultrasmall Iron Oxide Nanoparticles for Enhanced and Dynamic Magnetic Resonance Imaging of Arthritis

Xin Li, Shiyi Lu, Zuogang Xiong, Yong Hu, Dan Ma, Wenqi Lou, Chen Peng, Mingwu Shen,* and Xiangyang Shi**

Copyright WILEY-VCH Verlag GmbH & Co. KGaA, 69469 Weinheim, Germany, 2019.

Supporting Information

Light-Addressable Nanoclusters of Ultrasmall Iron Oxide Nanoparticles for Enhanced and Dynamic Magnetic Resonance Imaging of Arthritis

Xin Li, Shiyi Lu, Zuogang Xiong, Yong Hu, Dan Ma, Wenqi Lou, Chen Peng, Mingwu Shen*, Xiangyang Shi**

Mr. X. Li,[+] Miss S. Lu,[+] Mr.Y. Hu, Miss D. Ma, Prof. M. Shen, Prof. X. Shi
State Key Laboratory for Modification of Chemical Fibers and Polymer Materials, International Joint Laboratory for Advanced Fiber and Low-dimension Materials, College of Chemistry, Chemical Engineering and Biotechnology, Donghua University, Shanghai 201620, People's Republic of China
E-mail: mwshen@dhu.edu.cn (M. Shen) and xshi@dhu.edu.cn (X. Shi)

Mr. Z. Xiong, Mr. W. Lou, Dr. C. Peng
China-France Joint Laboratory for Healthcare Theranostics, and Cancer Center, Shanghai Tenth People's Hospital, Tongji University School of Medicine, Shanghai 200072, People's Republic of China
E-mail: pengchen_1985@163.com (C. Peng)

[+] Authors contributed equally to this work.

Keywords: Ultrasmall Fe_3O_4 NPs; light-addressable nanoclusters; T_1/T_2-weighted MR imaging; folic acid-mediated targeting; inflammatory arthritis

Experimental

Materials

Anhydrous ferric chloride ($FeCl_3$) and sodium citrate (Na_3Cit) were obtained from Sinopharm Chemical Reagent Co., Ltd. (Shanghai, China). Polyethylene glycol (PEG) with amine group at one end and amine group protected by t-butyloxy carbonyl at the other end (NH_2-PEG-NHBoc, M_w = 5000) were supplied by Shanghai Yanyi Biotechnology Corporation (Shanghai, China). Diethylene glycol, 1-ethyl-3-(3-dimethylaminopropyl) carbodiimide hydrochloride (EDC), N-hydroxysuccinimide (NHS), dimethylsulfoxide (DMSO), $CDCl_3$ and D_2O were acquired from J&K Chemical Reagent Co., Ltd. (Shanghai, China). NHS activated folic acid (NHS-FA) was obtained from Hunan Hua Teng Pharmaceutical Co., Ltd. (Changsha, China). Sodium acetate, levulinic acid, ammonia (7 N NH_3 in methanol), hydroxylamine-*O*-sulfonic acid, methanol, trimethylamine, iodine (I_2), ethyl acetate, hydrochloric acid (HCl), sodium thiosulfate, magnesium ($MgSO_4$) and dichloromethane (DCM) were purchased from Aldrich (St. Louis, MO). Raw 264.7 cells (mouse macrophage cells) were obtained from Institute of Biochemistry and Cell Biology, the Chinese Academy of Sciences (Shanghai, China). Cell Counting Kit-8 (CCK8) was purchased from 7Sea Biotech. Co., Ltd. (Shanghai, China). Dulbecco's Modified Eagle Medium (DMEM), penicillin, streptomycin, and fetal bovine serum (FBS) were supplied from Hangzhou Jinuo Biomedical Technology (Hangzhou, China).

Characterization techniques

^{1}H NMR and ^{13}C NMR spectra were collected on a Bruker AV400 nuclear magnetic resonance spectrometer using $CDCl_3$ or D_2O as solvent. Transmission electron microscopy (TEM) imaging was performed on a JEOL 2010F analytical electron microscope (JEOL, Tokyo, Japan) operating at a voltage of 200 kV. TEM samples were prepared by depositing a diluted particle suspension onto carbon-coated copper grid and air dried before imaging.

Thermal gravimetric analysis (TGA) was carried out on a TG 209 F1 thermogravimetric analyzer (NETZSCH Instruments Co., Ltd, Selb/Bavaria, Germany) with the heating rate of 10 $^{o}C\ min^{-1}$ under N_2 atmosphere. Dynamic light scattering (DLS) measurements were conducted using a Malvern Zetasizer Nano ZS model ZEN3600 (Worcestershire, UK) equipped with a standard 633 nm laser at room temperature. Leeman Prodigy inductively coupled plasma-optical emission spectroscopy (ICP-OES, Hudson, NH) was performed to analyze the Fe concentration in aqueous solution. T_1 and T_2 relaxometries were carried out on a 0.5 T NMI20-Analyzing and Imaging system (Shanghai NIUMAG Corporation, Shanghai, China) with an Fe concentration ranging from 0.1 to 1.6 mM. The parameters were set as follows: TR = 400 ms, TE = 20 ms, resolution = 156 mm × 156 mm, and section thickness = 0.5 mm for T_1 relaxometry, and TR = 3000 ms, TE = 60 ms, resolution = 156 mm × 156 mm, and section thickness = 0.5 mm for T_2 relaxometry. Bio-TEM images were observed using a TEM (Tecnai G2 Spirit, FEI) with the cells cutting into 75 nm thick slices according to protocols described in the literature.[1]

Synthesis of ultrasmall Fe_3O_4 NPs

Citric acid-stabilized ultrasmall Fe_3O_4 NPs were prepared by a solvothermal method according to the literature[2] with a slight modification. Typically, anhydrous $FeCl_3$ (4 mmol) was dissolved in diethylene glycol (40 mL) under ultrasonication to form a homogeneous solution. Na_3Cit (1.6 mmol) was added to the above solution, and the mixture was heated to 80 °C in a water bath to form a clear solution. Subsequently, sodium acetate (12 mmol) was added to the above mixture solution and then the mixture was transferred to a Teflon-lined stainless-steel autoclave with a volume of 50 mL and sealed in air. The autoclave was then placed in an oven at 200 °C for 5.5 h. After cooling down to room temperature, the black solution was collected by centrifugation (10 000 rpm, 10 min) and purified with ethanol (2 times) and water (1 time) to remove excess reactants and byproducts. The resulting black product was redispersed into water and lyophilized to obtain ultrasmall Fe_3O_4 NPs.

Synthesis of NHS-DA and NH_2-PEG-(DA)-FA

First, the light-responsive molecular switch of NHS-diazirine (DA) was synthesized according to the literature.[3] In brief, levulinic acid (2.0 g) in a round-bottom flask was cooled to 0 °C under N_2 protection and then ammonia solution (7 N NH_3 in methanol, 25 mL) was slowly added. After 3 h, the hydroxylamine-*O*-sulfonic acid (NH_2OSO_3H, 3.2 g, in 4 mL anhydrous methanol) was dropwise introduced. The resulting solution was allowed to warm to room temperature overnight under stirring, and concentrated under vacuum to form residues on the wall of flask, which were then resuspended in anhydrous methanol. The suspension was filtrated to remove the white precipitate, and the total filtrate was concentrated through reduced pressure evaporation and then redissolved in anhydrous methanol (50 mL). At 0 °C, triethylamine (TEA, 4.3 mL) was added, followed by addition of anhydrous methanol solution of I_2 (2.1 g, 2.5 mL) until a dark brown color appeared and persisted for more than 10 min. This indicates the complete deoxidation of the diaziridine intermediate. The solution was then diluted with ethyl acetate, washed with 1 M HCl and saturated aqueous solution of sodium thiosulfate. After that, the organic phase was separated and dried with anhydrous $MgSO_4$, followed by vacuum evaporation to yield the DA as pale-yellow oil (0.62 mg, 28%) after purification with silica gel flash chromatography. Then, 10 mL of anhydrous DCM solution containing DA (0.35 g), NHS (0.34 g) and EDC (0.57 g) was mixed under stirring at 0 °C. After being stirred at room temperature for 2 h, the reaction mixture was diluted with DCM, washed with water (3 times, 75 mL) and brine (1 time, 25 mL). The organic phase was dried with anhydrous $MgSO_4$, and concentrated under vacuum to obtain crude product, followed by purification with silica gel flash chromatography to obtain the NHS-DA. The schematic synthesis of NHS-DA is given as follows:

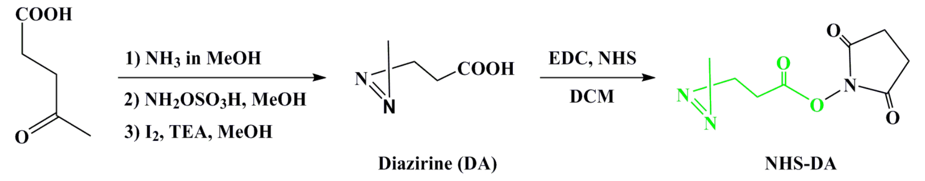

To prepare the mixed segments of NH_2-PEG-DA and NH_2-PEG-FA (for short, NH_2-PEG-(DA)-FA), 50 mg of NH_2-PEG-NHBoc dissolved in DMSO solution (5 mL) was stirred to form a homogeneous solution, then added with NHS-DA (5.6 mg, in 4 mL DMSO) and NHS-FA (13.7 mg, in 5 mL DMSO) simultaneously under stirring for 3 days. Then, 50 μL of HCl (3-4 M) was added to the above solution under stirring for 1-2 h to exfoliate the group of –Boc. The reaction mixture was then dialyzed against water (9 times, 2 L) using a dialysis membrane with a molecular weight cut-off (MWCO) of 1000 for 3 days. The dialysis liquid was freeze-dried to obtain the mixture of NH_2-PEG-(DA)-FA segments.

Synthesis of Fe_3O_4-PEG-(DA)-FA NPs

The ultrasmall Fe_3O_4 NPs (56 mg) dispersed in DMSO (5 mL) was activated by EDC (144 mg, in 2 mL DMSO) under vigorously stirring for 30 min, then NHS (70 mg, in 1 mL DMSO) was added into the above solution under stirring for another 3 h. After that, the NH_2-PEG-(DA)-FA solution (7 mg, in 3 mL DMSO) was dropwise added to the above solution under vigorous stirring for 3 days. The reaction mixture was then dialyzed against water (9 times, 2 L) using a dialysis membrane with an MWCO of 5000 for 3 days, followed by lyophilization to obtain the product of Fe_3O_4-PEG-(DA)-FA NPs.

Light-responsiveness and T_1/T_2 MR relaxometry of the Fe_3O_4-PEG-(DA)-FA NPs

The assembly kinetics and corresponding T_1/T_2 relaxation rates of the Fe_3O_4-PEG-(DA)-FA NPs were investigated, respectively. The aqueous solution of Fe_3O_4-PEG-(DA)-FA NPs ([Fe] = 3 mM, 100 μL) was irradiated under a 405 nm laser (1.0 W cm^{-2}) for different time periods (0, 2, 5, 8 and 12 min, respectively). The morphology and hydrodynamic size of the Fe_3O_4-PEG-(DA)-FA NPs and Fe_3O_4-PEG-(DA)-FA nanoclusters (NCs) with different sizes were characterized by TEM and DLS, respectively. The corresponding T_1/T_2 relaxation rates and T_1/T_2-weighted MR imaging of Fe_3O_4-PEG-(DA)-FA NPs and Fe_3O_4-PEG-(DA)-FA NCs under laser irradiation for different time periods were tested using a 0.5 T NMI20-Analyst

NMR analyzing and imaging system. According to the theory and equation,[4] the r_1, r_2 and the ratio of r_2/r_1 was calculated to evaluate T_1 and T_2 effect of Fe_3O_4-PEG-(DA)-FA NPs and Fe_3O_4-PEG-(DA)-FA NCs.

Cell culture

Raw264.7 cells were cultured and passaged in fresh DMEM with 10% FBS, 100 U/mL penicillin and 100 U/mL streptomycin at 37 ^oC in a 5% CO_2 incubator. The Raw264.7 cells cultured in free FA-containing medium (2 mM) overnight were identified as free FA-blocked Raw264.7 cells, while Raw264.7 cells without specific declaration represent normal Raw264.7 cells.

In vitro cytotoxicity assay, cellular uptake and light-triggered assembly of Fe_3O_4-PEG-(DA)-FA NPs within cells

Cell Counting Kit-8 (CCK8) assay was used to evaluate the cytotoxicity of the Fe_3O_4-PEG-(DA)-FA NPs at different Fe concentrations (0-3.0 mM). Raw264.7 cells were seeded into a 96-well plate at a density of 1.0×10^4 cells/well with fresh DMEM the day before the experiment. Then the medium in each well was replaced with fresh medium (100 μL) containing PBS (10 μL) or Fe_3O_4-PEG-(DA)-FA NPs (10 μL) with the final Fe concentration ranging from 0 to 3.0 mM. The cells were incubated at 37 ^oC and 5% CO_2 for another 24 h. Then the cells were washed 3 times with PBS, and the CCK8 (10 μL) was added into each well with fresh medium (100 μL) and the cells were incubated continuously for another 3 h. Finally, the absorbance at 450 nm in each well was recorded using a Multiskan MK3 ELISA reader (Thermo Scientific, Waltham, MA).

To investigate the FA-mediated targeted cellular uptake of the Fe_3O_4-PEG-(DA)-FA NPs, Raw264.7 and free FA-blocked Raw264.7 cells were respectively incubated with the particles at different Fe concentrations (0-3.0 mM). In brief, the cells were seeded at a density of 4×10^6 cells in a 25 cm^2 culture flask with 3 mL regular DMEM. After overnight culture, the medium was replaced with fresh medium (3 mL) containing PBS (300 μL) or the Fe_3O_4-PEG-

(DA)-FA NPs (300 μL) at different Fe concentrations (0-3.0 mM), and the cells were incubated at 37 °C and 5% CO_2 for an additional 6 h. Subsequently, the cells were washed for 3 times with PBS, lifted with trypsinization, centrifuged, and resuspended in PBS. The cells were counted and lysed using an aqua regia solution (1 mL) to digest both the cells and the particles. Each sample was diluted with 2 mL of water before quantification of the Fe concentration using ICP-OES.

Further, the specific cellular uptake of the Fe_3O_4-PEG-(DA)-FA NPs in the Raw264.7 and free FA-blocked Raw264.7 cells, and light-triggered assembly of Fe_3O_4-PEG-(DA)-FA NPs in the Raw264.7 cells under laser irradiation for 3 min were observed using qualitative Bio-TEM. Raw264.7 and free FA-blocked Raw264.7 cells were separately seeded into a 12-well plate at a density of 2.5×10^5 cells/well with 1 mL of fresh DMEM and cultured overnight. Then, the medium was replaced with fresh medium (1 mL) containing the Fe_3O_4-PEG-(DA)-FA NPs with the final Fe concentration of 3 mM and the cells were incubated for 12 h at 37 °C and 5% CO_2. After that, the cells were washed 3 times with PBS, then cells incubated with Fe_3O_4-PEG-(DA)-FA NPs were exposed to 405 nm laser (1.0 W cm^{-2}) for 3 min, and cells that were not laser irradiated served as control. Subsequently, the cells were lifted with trypsinization, centrifuged, resuspended in PBS containing 2.5% glutaraldehyde and fixed at 4 °C. Last, the samples were post-processed according to protocols described in the literature,[1] and the cell sections with a thickness of 75 nm were observed using Bio-TEM according to the literature.[5]

In vivo T_1/T_2-weighted MR imaging of inflammatory arthritis

Animal experiments were carried out following the protocols approved by the institutional committee for animal care and the policy of the National Ministry of Health. The 5-week old male nude mice (22-25 g) were purchased from Shanghai Slac Laboratory Animal Center (Shanghai, China). The ligamentum in the knee joint of right or two hind leg for each mouse was cut *via* a surgery method,[6] and the arthritis model was established after one week.

The mice were randomly divided into each each group (n = 3). For T_1-weighted MR imaging, the Fe_3O_4-PEG-(DA)-FA NPs dispersed in 200 μL of saline ([Fe] = 30 mM) were intravenously injected to each mouse bearing arthritis in its right hind leg via tail vein. To validate the targeting specificity of the Fe_3O_4-PEG-(DA)-FA NPs to arthritis, free FA (6 mM, 100 μL) was first intravenously injected to each mouse to obtain the free FA-blocked arthritis model, followed by subsequent intravenous injection of the NPs. Then, 2D spin-echo T_1-weighted or T_2-weighted MR imaging of arthritis *in vivo* was carried out using a 3.0-T Signa HDxt superconductor clinical MR system (Siemens Healthcare, Erlangen, Germany) under the following parameters: TR/TE = 1200/10.7, matrices = 256 × 256, FOV = 12 mm, bandwidth = 140 Hz Px^{-1}, slice thickness = 0.9 mm for T_1-weighted MR imaging; TR/TE = 7500/77, matrices = 256 × 256, FOV = 9 mm, bandwidth = 140 Hz Px^{-1}, slice thickness = 0.9 mm for T_2-weighted MR imaging. The images were collected before injection and at 15, 30, 45, 60, 90, and 120 min postinjection. For T_1/T_2-weighted MR imaging, the Fe_3O_4-PEG-(DA)-FA NPs were injected to each mouse bearing arthritis in its two hind legs *via* tail vein ([Fe] = 30 mM, in 200 μL saline for each hind leg). Then, the 405 nm laser irradiation (1.0 W cm^{-2}) for 12 min on the left hind leg was performed at 30 min postinjection. Note that 30 min was the peak time of Fe_3O_4-PEG-(DA)-FA NPs accumulation in the arthritis. In contrast, the right hind leg received no laser irradiation. T_1-weighted and T_2-weighted MR imaging were performed before injection (control group), before and after laser irradiation, respectively.

Histological examination of the particle uptake in inflammation region of arthritis

The Safranin O, H&E and Prussian blue staining were carried out to determine the histopathological changes and to analyze the retention of the Fe_3O_4-PEG-(DA)-FA NCs in the inflammatory arthritis region of the mice, respectively. The arthritis-bearing mice were randomly divided into six groups with five mice in each group: Saline, NPs + Free FA-blocked, NPs, Laser-NCs, NPs + 2 h and Laser-NCs + 2 h. For Saline or NPs groups, saline

(200 μL) or Fe_3O_4-PEG-(DA)-FA NPs dispersed in saline ([Fe] = 30 mM, 200 μL) were intravenously injected to each mouse. For NPs + Free FA-blocked, each mouse was first injected with free FA (6 mM, 100 μL in saline) and then with saline containing Fe_3O_4-PEG-(DA)-FA NPs ([Fe] = 30 mM, 200 μL). For Laser-NCs group, at 30 min postinjection, laser irradiation (405 nm, 1.0 W cm^{-2}) was performed at arthritis region for 12 min. After that, the arthritis tissue in the groups of Saline, NPs + Free FA-blocked, NPs and Laser-NCs were obtained simultaneously. After 2 h, the arthritis tissue in the groups of NPs + 2 h and Laser-NCs + 2 h were also extracted. Then, the sections of arthritis in the knee joint region were prepared and post-processed for three types of histological examinations according to literature protocols.[6-7] The stained arthritis sections were observed using a Leica DM IL LED inverted phase contrast microscope with a magnification of 200× for each sample. Meanwhile, the normal mice treated with saline (Normal + Saline) and Fe_3O_4-PEG-(DA)-FA NPs (Normal + NPs) were used as the control groups.

Statistical Analysis

One-way analysis of variance (ANOVA) statistical method was performed to evaluate the experimental data. A value of 0.05 was selected as the significance level and the data were indicated with (*) for $p < 0.05$, (**) for $p < 0.01$, and (***) for $p < 0.001$, respectively.

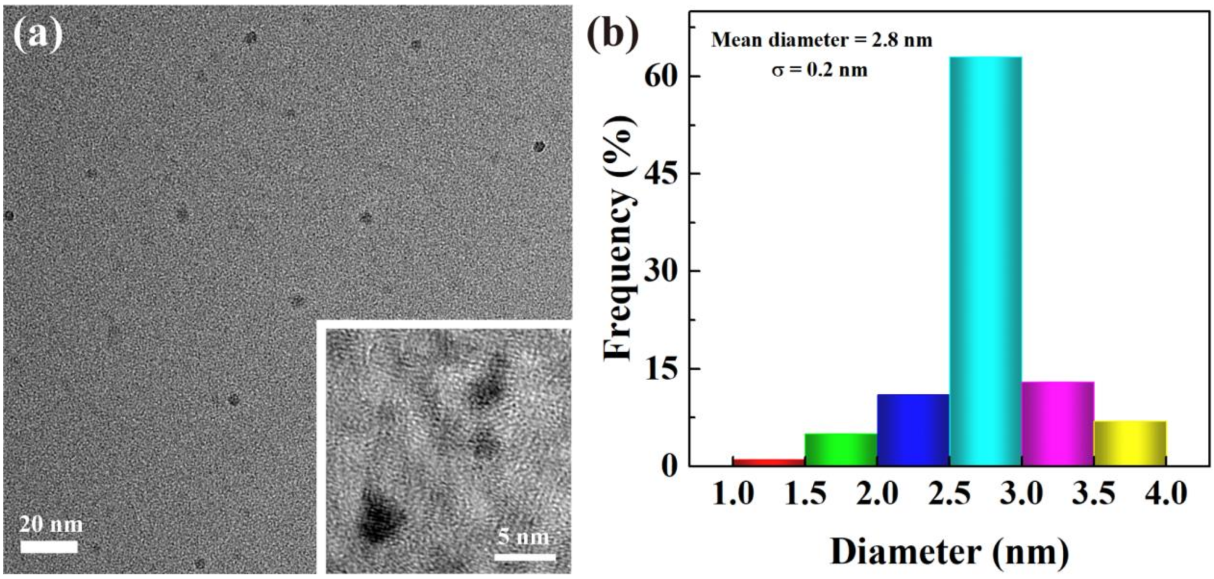

Figure S1. (a) TEM images and (b) size distribution histogram of citric acid-stabilized ultrasmall Fe_3O_4 NPs.

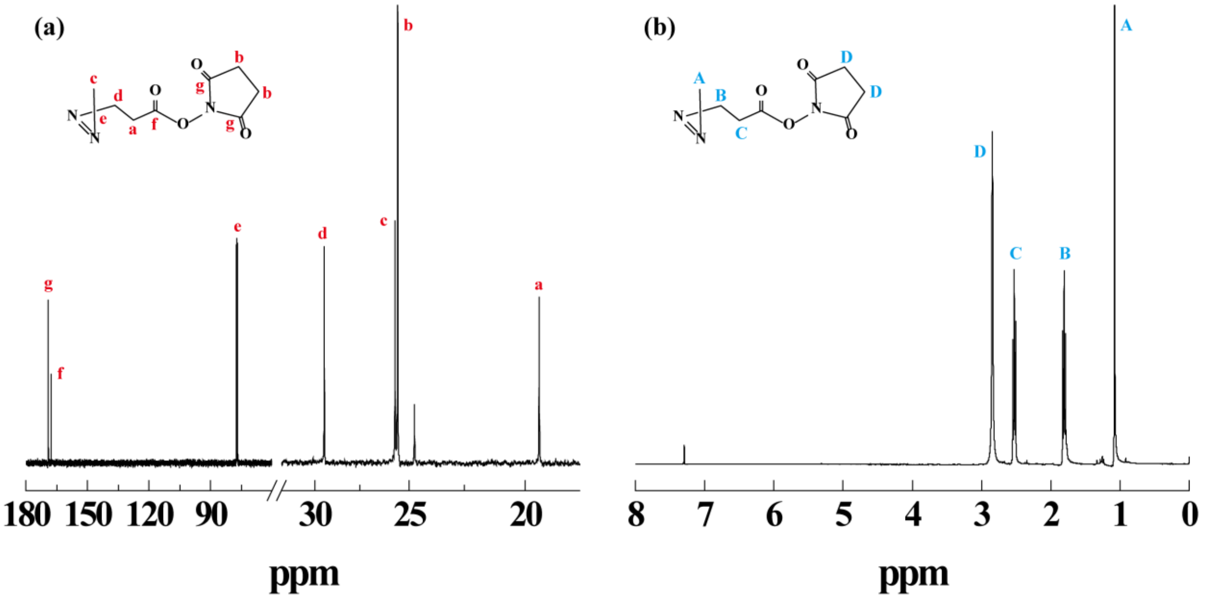

Figure S2. (a) ^{13}C NMR and (b) ^{1}H NMR spectra of NHS-DA dispersed in $CDCl_3$.

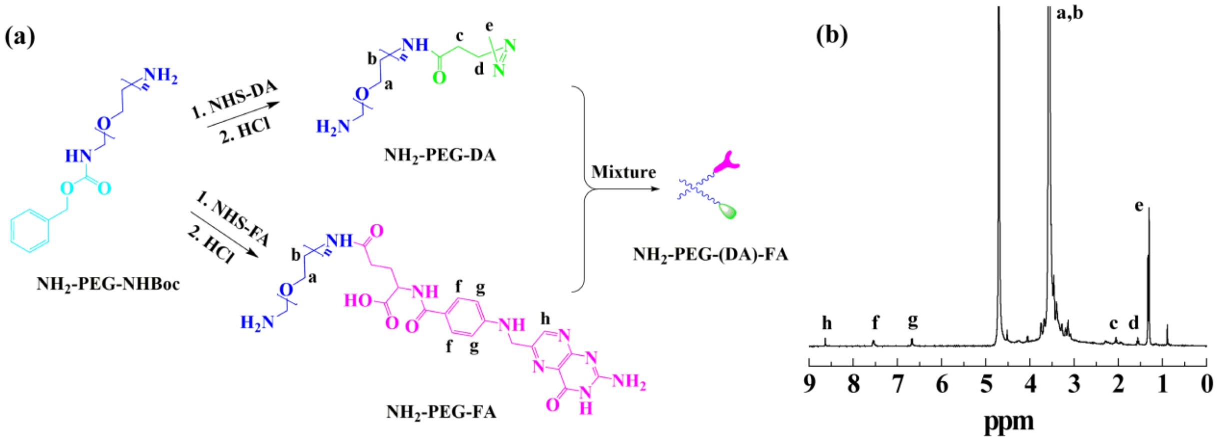

Figure S3. (a) Schematic illustration of the synthesis of NH_2-PEG-(DA)-FA and (b) ^{1}H NMR spectra of NH_2-PEG-(DA)-FA dissolved in D_2O.

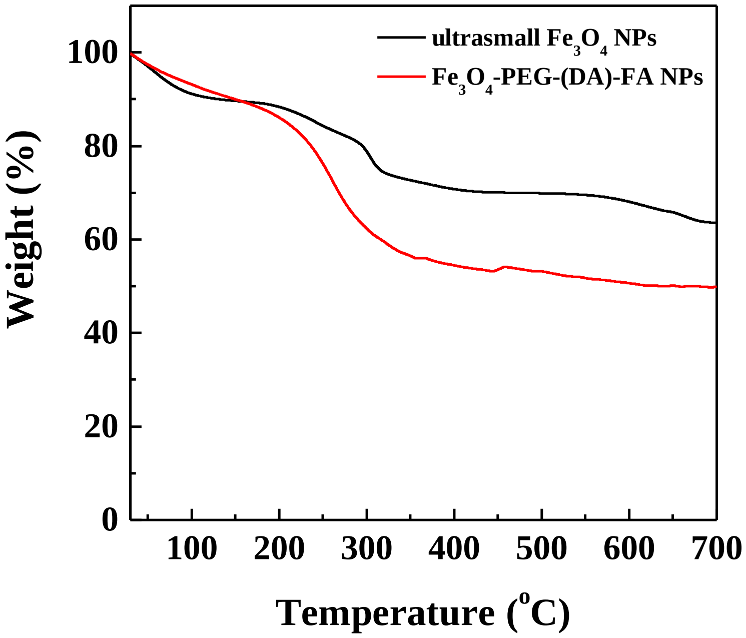

Figure S4. TGA curves of ultrasmall Fe_3O_4 and Fe_3O_4-PEG-(DA)-FA NPs.

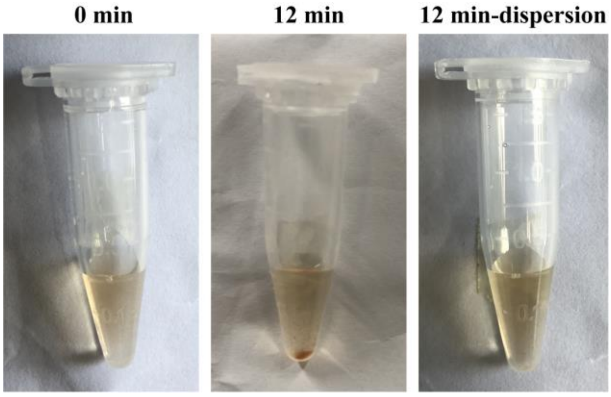

Figure S5. The digital photos of the Fe_3O_4-PEG-(DA)-FA NPs before laser irradiation, the aggregated and dispersed Fe_3O_4-PEG-(DA)-FA NCs after laser irradiation for 12 min.

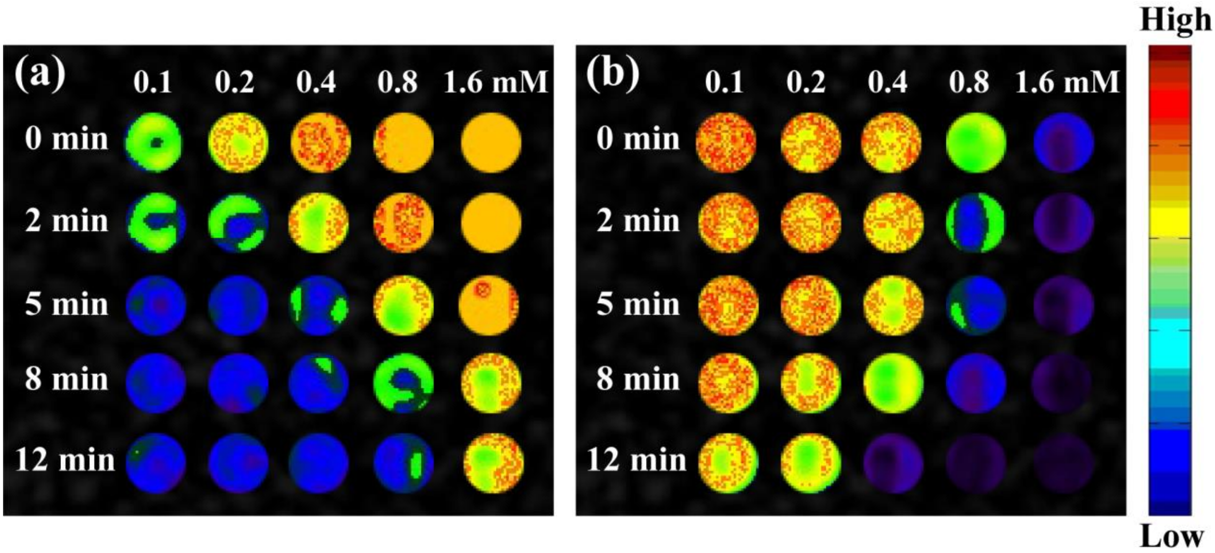

Figure S6. In vitro color T_1-weighted (a) and T_2-weighted (b) MR images of Fe_3O_4-PEG-(DA)-FA NPs and Fe_3O_4-PEG-(DA)-FA NCs (at different Fe concentrations) under 405 nm laser irradiation (1.0 W cm^{-2}) for different time periods.

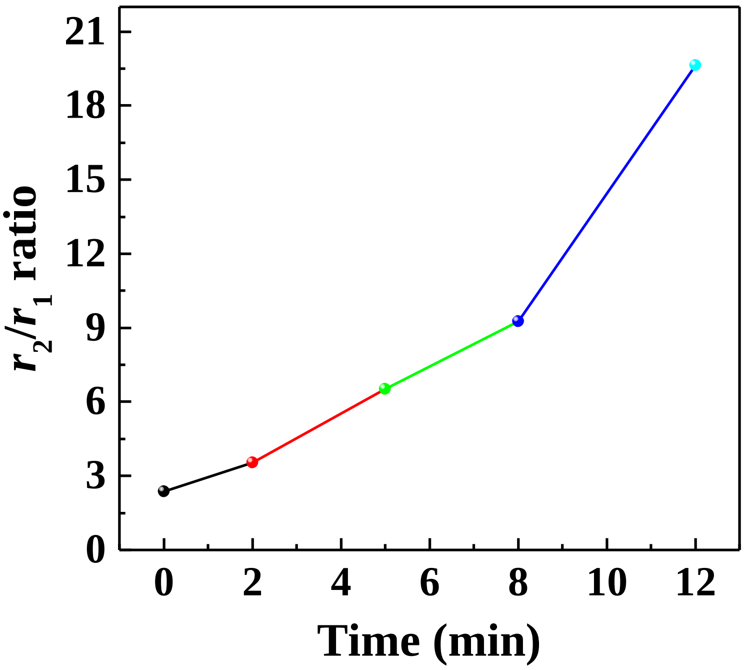

Figure S7. The r_2/r_1 ratios of the Fe_3O_4-PEG-(DA)-FA NPs and Fe_3O_4-PEG-(DA)-FA NCs under 405 nm laser irradiation (1.0 W cm^{-2}) for different time periods.

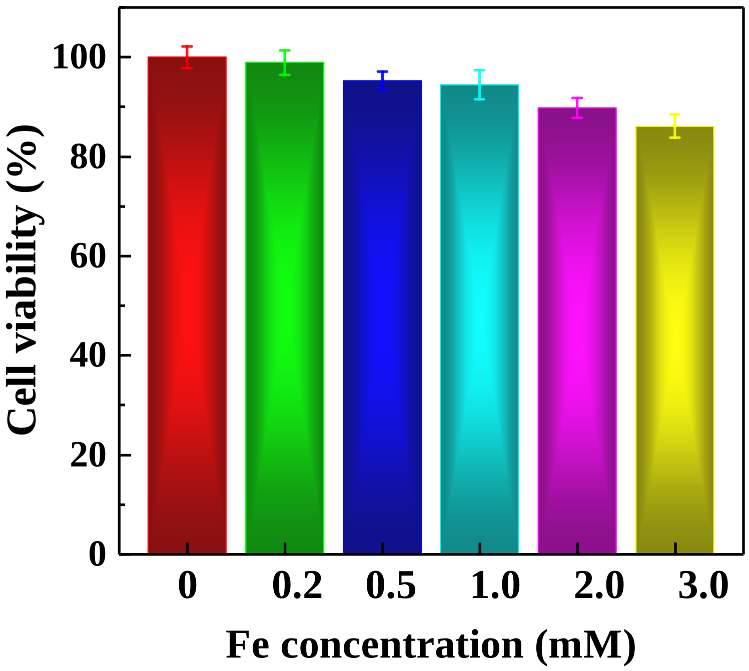

Figure S8. CCK8 viability assay of Raw264.7 cells treated with the Fe_3O_4-PEG-(DA)-FA NPs at different Fe concentrations for 24 h. The cells treated with PBS were used as control (100%).

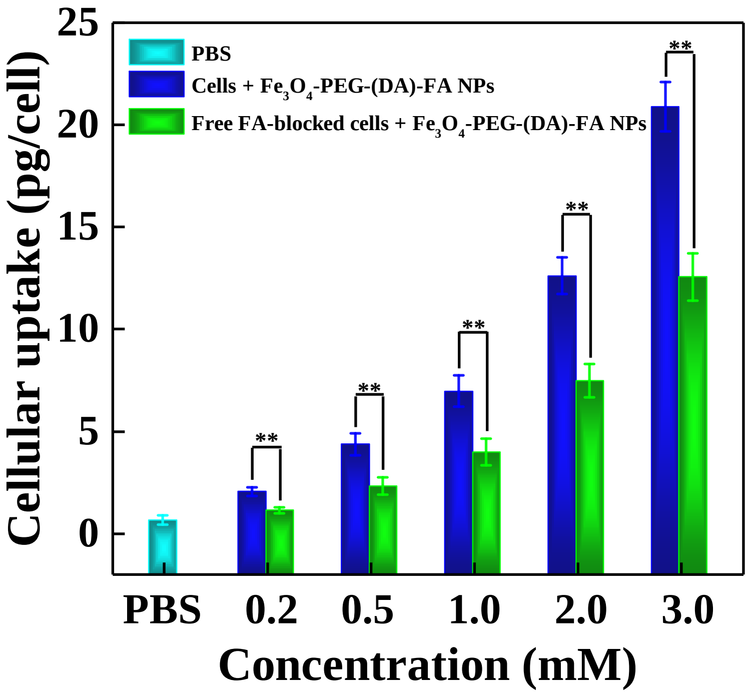

Figure S9. Fe uptake in Raw264.7 cells and free FA-blocked Raw264.7 cells after they were treated with the Fe_3O_4-PEG-(DA)-FA NPs at different Fe concentrations for 6 h. The cells treated with PBS were used as control.

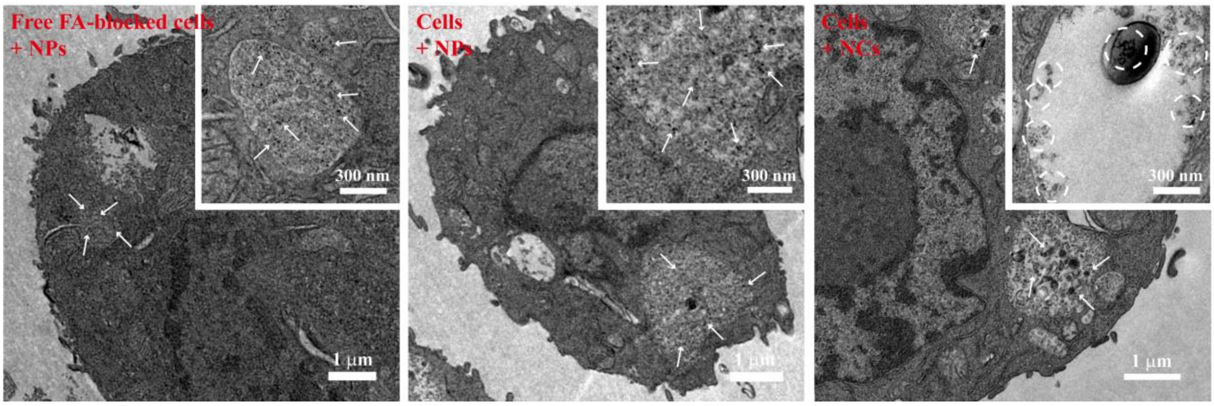

Figure S10. TEM images of free FA-blocked Raw264.7 cells and Raw264.7 cells treated with the Fe_3O_4-PEG-(DA)-FA NPs at the Fe concentration of 3 mM without or with 405 nm laser irradiation for 3 min.

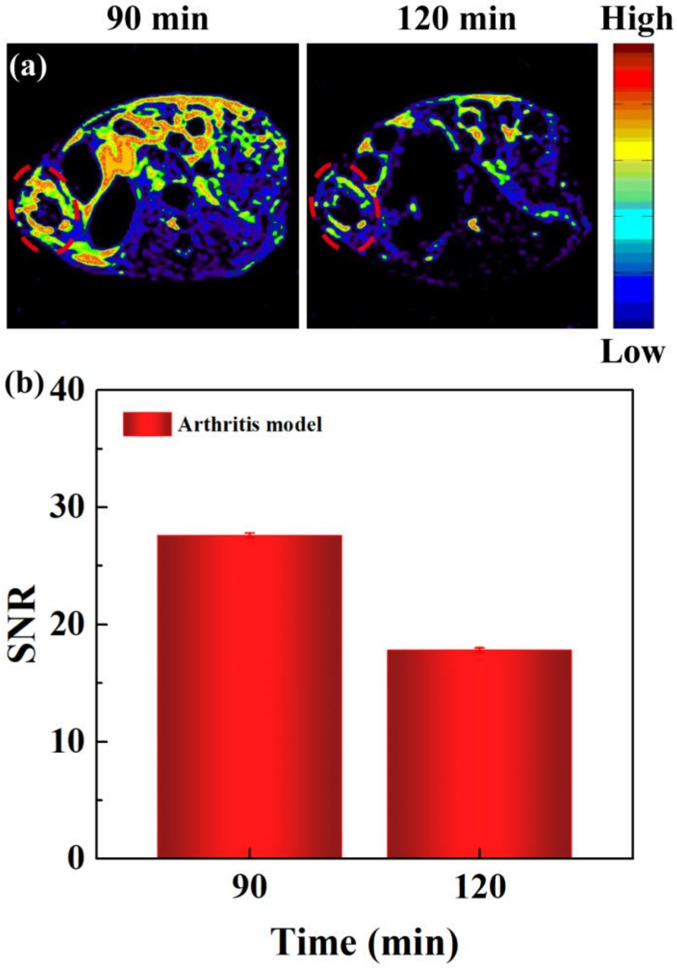

Figure S11. *In vivo* (a) T_1-weighted MR imaging and (b) corresponding MR SNR of arthritis model after intravenous injection of Fe_3O_4-PEG-(DA)-FA NPs for 90 and 120 min. The dashed red circles indicated the arthritis region.

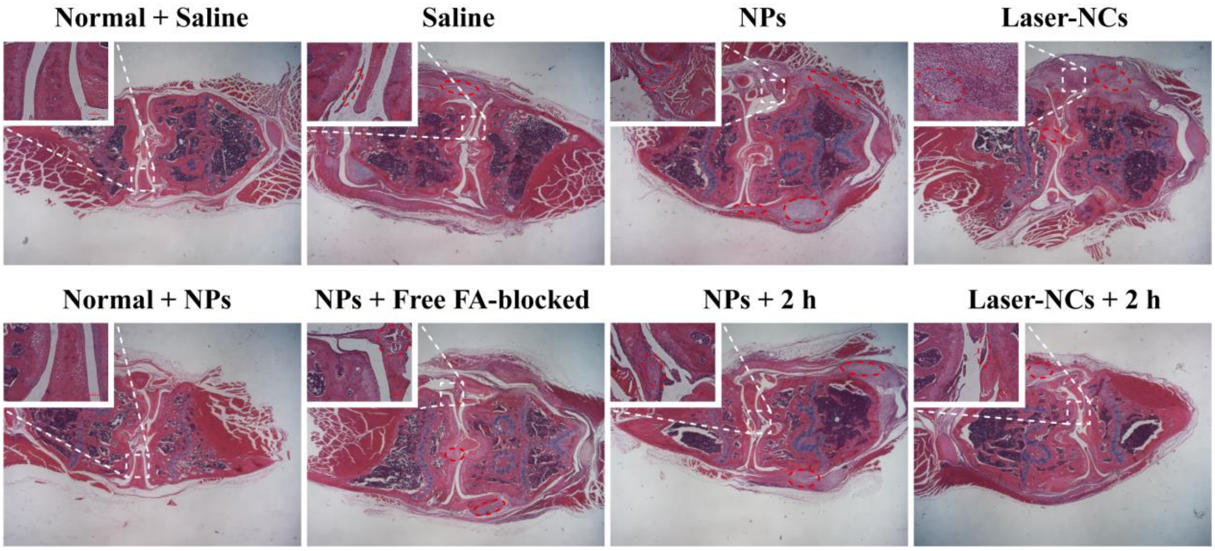

Figure S12. H&E stained tissue sections of arthritis region after different treatments.

References

[1] S. H. Wang, X. Shi, M. Van Antwerp, Z. Cao, S. D. Swanson, X. Bi and J. R. Baker, *Adv. Funct. Mater.* **2007**, *17*, 3043-3050.
[2] D. Ma, J. W. Chen, Y. Luo, H. Wang and X. Y. Shi, *J. Mater. Chem. B* **2017**, *5*, 7267-7273.
[3] T. Kambe, B. E. Correia, M. J. Niphakis and B. F. Cravatt, *J. Am. Chem. Soc.* **2014**, *136*, 10777-10782.
[4] a) J. F. Zeng, L. H. Jing, Y. Hou, M. X. Jiao, R. R. Qiao, Q. J. Jia, C. Y. Liu, F. Fang, H. Lei and M. Y. Gao, *Adv. Mater.* **2014**, *26*, 2694-2698; b) S. Laurent, D. Forge, M. Port, A. Roch, C. Robic, L. V. Elst and R. N. Muller, *Chem. Rev.* **2008**, *108*, 2064-2110.
[5] X. Li, Z. Xiong, X. Xu, Y. Luo, C. Peng, M. Shen and X. Shi, *ACS Appl. Mater. Interfaces* **2016**, *8*, 19883-19891.
[6] Y. O. Son, S. Park, J. S. Kwak, Y. Won, W. S. Choi, J. Rhee, C. H. Chun, J. H. Ryu, D. K. Kim, H. S. Choi and J. S. Chun, *Nat. Commun.* **2017**, *8*, 2133.
[7] a) M. F. Falangola, S. P. Lee, R. A. Nixon, K. Duff and J. A. Helpern, *Neurochem. Res.* **2005**, *30*, 201-205; b) J. Jeong, K. Bae, S. G. Kim, D. Kwak, Y. J. Moon, C. H. Choi, Y. R. Kim, C. S. Na and S. J. Kim, *BMC Complementary Altern. Med.* **2018**, *18*, 131.
